# Diode laser versus sclerotherapy: bloodless approaches in the treatment of oral pyogenic granuloma (randomised controlled clinical trial)

**DOI:** 10.1007/s10266-022-00759-9

**Published:** 2022-10-28

**Authors:** Souzy Kamal Anwar, Sandra Nabil Edward, Naguiba Mahmoud ELSayed

**Affiliations:** Oral Medicine, Periodontology, Oral Diagnosis and Oral Radiology Department, Faculty of Dentistry, Alexandria University, Alexandria, 21521 Egypt

**Keywords:** Diode laser, Ethanolamine oleate, Oral pyogenic granuloma, Sclerotherapy

## Abstract

Oral pyogenic granuloma (PG) is traditionally treated by surgical excision which is associated with bleeding, pain and a high rate of recurrence. Our research aimed to clinically assess the effectiveness of diode laser versus sclerotherapy, as bloodless approach, in the treatment of oral PG. We randomly divided 20 patients with oral PG into two groups, with those in the test group being managed via diode laser application and those in the control group via injections of ethanolamine oleate as a sclerosing agent. All patients were evaluated intraoperatively for bleeding severity and postoperatively for pain. The quality of healing was also assessed using Landry healing index after the 1st, 2nd and 4th weeks. Additionally, the patients were recalled after 3, 6 and 9 months from the end of treatment for recurrence evaluation. Our results revealed that intraoperative bleeding did not differ significantly between both groups while postoperative pain decreased significantly in the sclerotherapy group compared to the laser group. For different intervals, the sclerotherapy group had a higher healing quality index than the laser group, although the difference was not statistically significant. However, recurrence occurred in the laser group, there were no cases of recurrence in the sclerotherapy group in all intervals. In conclusion, diode laser treatment of PG is a reliable, less invasive, and sensitive procedure that requires an experienced operator and specialised equipment. However, ethanolamine oleate sclerotherapy is an inexpensive, simple technique besides being less prone to recurrence problems, especially when treatment duration is not a concern.

## Introduction

Pyogenic granuloma (PG) is considered one of the most common forms of reactive hyperplasia that develops as a result of chronic tissue trauma that triggers a repair response [1]. It can occur on the skin or mucous membranes [2]. Intraorally, it can occur at any site; however, it most commonly affects the gingiva, followed by the lips, buccal mucosa and tongue [3, 4].

Although the etiopathogenesis of PG is unclear, various factors appear to have a role in its development [5]. It is considered a reactive lesion generated by a variety of stimuli, including trauma, chronic low-grade irritation and hormonal variables [3, 4]. It has also been linked to a few drugs, including oral contraceptives, retinoids, gefitinib, cabecitabine and afatinib [6–9].

There are numerous differential diagnoses for PG, therefore biopsy findings are critical and conclusive in establishing the diagnosis [4].

Many treatment modalities for PG have been introduced. Surgical excision is considered the treatment of choice; however, simple excision is associated with a relatively high rate of recurrence [10]. Therefore, excision with a margin of 2 mm around the clinical periphery (and to the periosteum), followed by curettage of underlying tissues with removal of the causative agent, must be performed to avoid recurrence [11]. Cryosurgery [12], electric cauterisation [13], sclerosing agents [14], intralesional steroids injections [15], neodymium-doped yttrium aluminium garnet (Nd: YAG) laser [16], erbium-doped yttrium aluminium garnet (Er: YAG) laser [17] and diode lasers [18] have also been used as viable substitutes for surgical excision.

Despite various treatment options, recurrence of PG is frequent, especially after normal surgical excision [5]. Recurrence happens following deficient excision, failure to eliminate etiologic factors or reinjury of lesions [1]. In the case of pregnancy, recurrence is common [5]. According to Vilmann et al. [2] cases of gingiva show a much greater recurrence rate than lesions from other mucosal sites of the oral cavity. In these cases, re-excision may be compulsory. Therefore, the adoption of deterrent methods, such as the use of a soft toothbrush, maintenance of better oral hygiene and patient follow-up is important in preventing the recurrence of PG [5, 19].

Diode lasers have been used as an alternative to surgical treatment for PG because of their benefits such as haemostasis, easier gingival reshaping, decreased postoperative pain and oedema in addition to its safety near calcified tissues due to its poor absorption by teeth and bones [20]. It has demonstrated excellent results in the excision of cutaneous PGs with few complications [18]. In terms of surgery duration and postoperative parameters, studies showed that diode laser irradiation more effectively induced complete epithelial photoablation and improved surgical comfort than surgical excision in the management of oral PG [21].

Sclerotherapy involves injecting a chemical agent into the vessels, which causes endothelial damage, thrombosis and vessels fibrosis, leading to lesion destruction [22]. As a result, it has been suggested that it could be a useful treatment modality for PG, which is made up of highly vascularised connective tissues [23]. It has been proposed as a good treatment choice when the lesion is either large or develops in an inaccessible area [24].

Ethanolamine oleate (EO), as a sclerosing agent, is a safe and effective treatment for benign vascular lesions located all over the body [25]. The oleic acid portion initiates coagulation while the ethanolamine portion organises fibrin clot suppression. As a result, the vascular lesion is replaced by fibrosis, which prevents further bleeding [26]. It has been also used successfully in the treatment of PG at three different concentrations (1.25, 2.5 and 5%) [27–29].

Traditional PG treatment approach by surgical excision, can be costly, invasive and require an office visit or operative suite setting [30]. In practice nowadays, alternative noninvasive treatment options are vital considerations for patients who have difficulty tolerating invasive therapies, especially young children and individuals with intellectual disabilities, procedural anxiety, or behavioral concerns [31].

Although recent studies confirmed the efficacy of sclerotherapy, as well as laser, being noninvasive treatment approaches compared to conventional invasive surgical excision of PG [21, 32, 33]. The literature on these noninvasive treatment approaches is very limited and includes only case reports and observational clinal studies. Comparisons between these bloodless noninvasive treatment modalities have been rarely investigated, and the best noninvasive treatment modality is yet to be figured out. Hence, we aimed to investigate the effectiveness of the diode laser in comparison to sclerotherapy on postoperative sequelae, the prognosis of healing and the recurrence rate in the treatment of oral PG.

The null hypothesis was that there would be no significant difference in post-operative complications, healing quality index and recurrence rate following treatment of oral pyogenic granuloma with diode laser in comparison with sclerotherapy.

## Materials and methods

### Study design

We conducted a two-arm parallel randomised controlled clinical trial on twenty patients with oral PG. A convenience sample of patients was recruited from the outpatient clinic of the Department of Oral Medicine, Periodontology, Diagnosis and Oral Radiology, Faculty of Dentistry, Alexandria University, Egypt. The study was conducted during the period between December 2020 and December 2021, after obtaining ethical approval from the Research Ethics Committee of the Faculty of Dentistry, Alexandria University, Egypt (IRB NO:00010556-IORG 0,008,839). This study was registered in the U.S National Institutes of Health Clinical Trials Registry (NCT05099081). It was also performed in accordance with the principles of the modified Helsinki code for human clinical studies, as revised in 2013 [34], and the CONSORT guidelines for reporting randomised clinical trials [35]. Written informed consent was obtained from each participant.

### Inclusion and exclusion criteria

We included patients aged 19–50 years of both genders who were diagnosed clinically, with confirmed histological confirmation, with gingival oral PG. The sizes of participants’ lesions were not less than 7 mm. Patients were excluded if they had uncontrolled diabetes, renal diseases, coagulation disorders or were allergic to any of the sclerosing drug constituents. Immune-compromised patients and pregnant and lactating women were also excluded from the study.

### Sample size estimation

The sample size was estimated based on a 5% alpha error and study power of 80%. The reported percentage of patients with excellent healing according to Landry’s healing index after three weeks of follow-up for the sclerotherapy group was 100% while it was 42.9% for the laser group [36]. The minimum calculated sample size was nine patients per group, which was increased to 10 patients per group to make up for any possible loss to follow-up. The total sample size = number of groups × number per group (2 × 10) = 20 patients. The sample size was based on Rosner’s method [37] calculated using G*power 3.0.10 [38].

### Grouping and randomisation

Simple randomisation of twenty patients diagnosed with oral PG into two groups (the laser [test] and sclerotherapy [control] groups) was carried out using a computer-generated list of random numbers (https://www.sealedenvelope.com/simple-randomiser/v1/lists). The list was generated using a random allocation software program. Each allocation was represented by a code and sealed in sequentially numbered opaque envelopes that were opened at the time of the intervention. Double-blinding was not applicable because of the nature of the study. However, the outcome assessor was blinded.

### Intervention

Before starting treatment (preoperatively), we obtained a complete medical and dental history from all the patients in both groups to figure out the size, texture, consistency, pain, location and duration of the lesion. We then performed phase I therapy and instructed patients to maintain oral hygiene measures [39].

Small incisional biopsy samples 2 × 2 mm were taken under local anaesthesia from the most ulcerated area and sent for histological analysis to confirm the diagnosis of the clinical picture. The tissue samples were fixed in 10% neutral formalin and embedded in paraffin. Five-micrometre sections were obtained and submitted for routine hematoxylin and eosin staining [40].

**Fig. 1:**
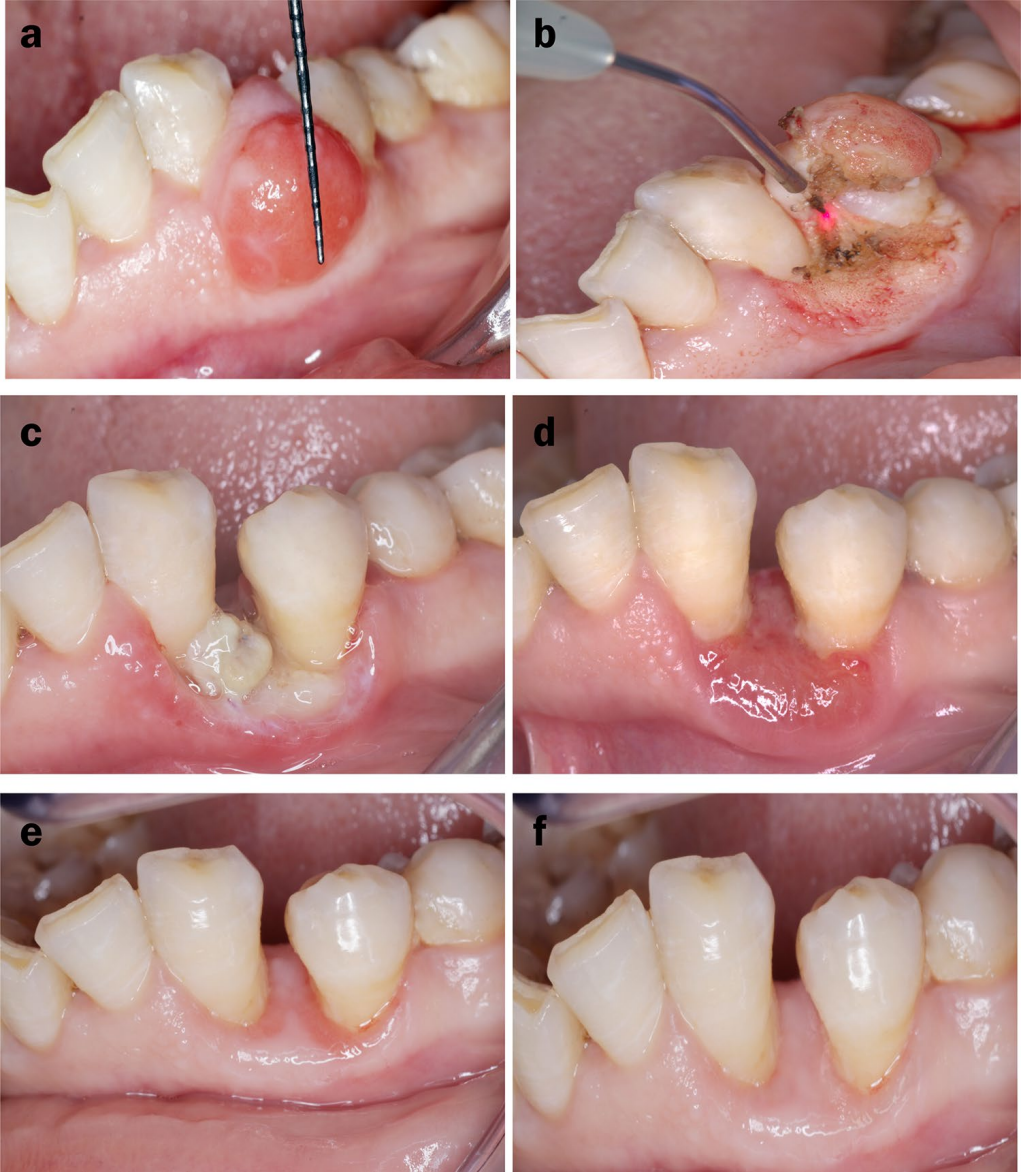
Clinical photographs showing a laser-treated case. **a** Measuring the size of the lesion (length) using periodontal probe. **b** Removal of the pyogenic granuloma using a laser tip. **c** Tissue healing after 1st week. **d** Tissue healing after 2nd week. **e** Tissue healing after 4th week. **f** Tissue healing at the 3rd month follow-up time point

After histopathological confirmation of PG, we divided the twenty patients into two groups as follows:

Laser-treated group (test): This group was made up of ten patients treated using diode lasers (Medency Primo, Piazza della Libertà, 49, 36,077 Altavilla Vicentina VI, Italy). The surgical area was anaesthetised using a local anaesthetic agent (Alexandricaine 1/100,000, Alexandria Co. for Pharmaceuticals & Chemical Industries, Awayed Alexandria, Egypt). Diode lasers (with a wavelength of 980 nm) were operated in the continuous wave mode with an output power of 3 W in the contact mode. The tip was moved around the base of the lesion in circles. The base of the lesion was cut precisely till the whole mass was entirely excised [33, 41]. (Fig. 1a,b).

**Fig. 2:**
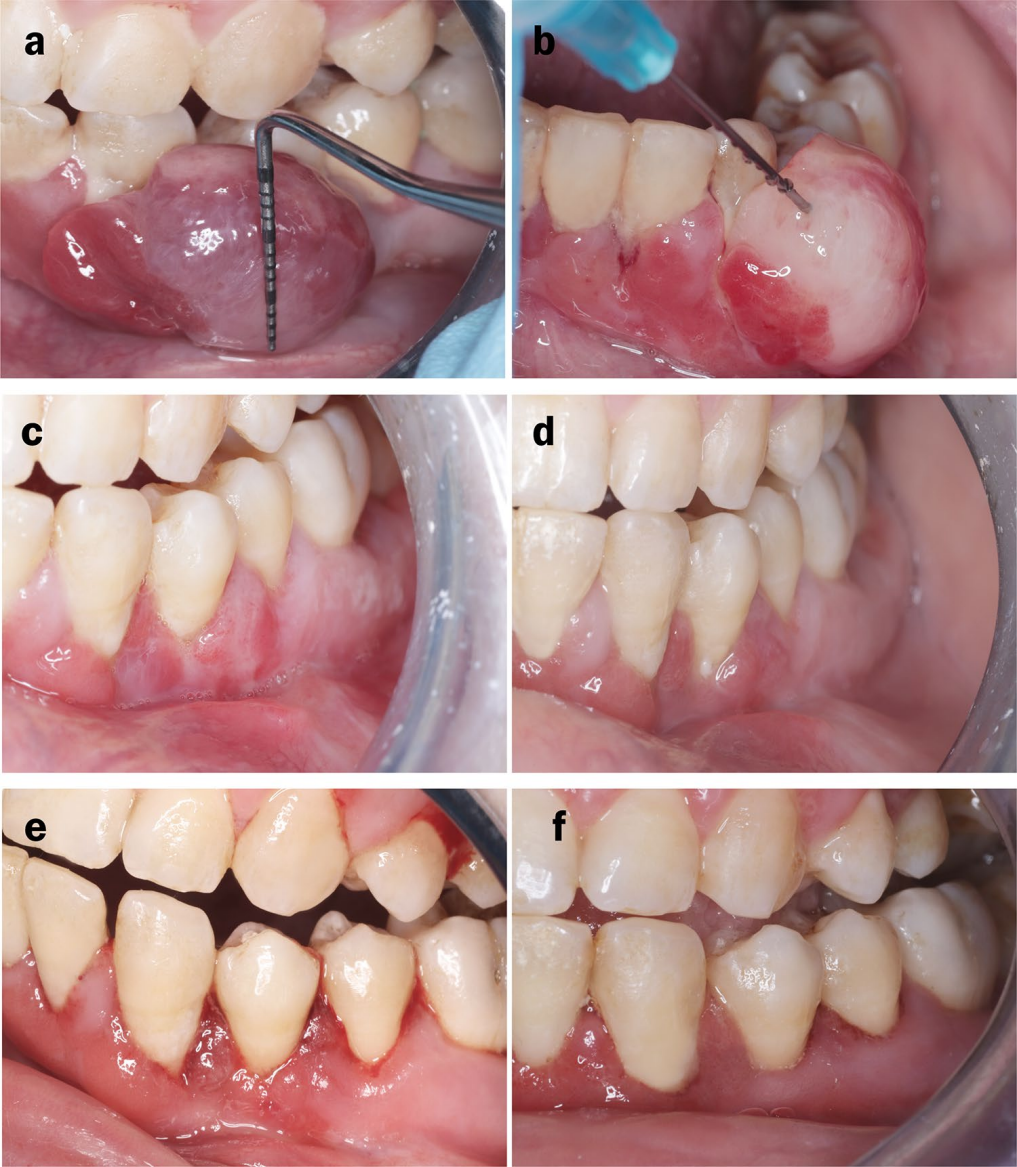
Clinical photographs showing a sclerotherapy-treated case. **a** Measuring the size of the lesion (length) using a periodontal probe. **b** Injection of the lesion with the sclerosing agent. **c** Tissue healing after 1st week. **d** Tissue healing after 2nd week. **e** Tissue healing after 4th week. **f** Tissue healing at the 3rd month follow-up time point

Sclerotherapy-treated (control) group: This group was made up of ten patients who were treated with injections of a sclerosing agent (Ethanolamine oleate® 5% Amp, EPICO, Egypt). After anaesthetising the surgical site with a local anaesthetic agent, the lesion was injected with the sclerosing agent, 5% EO, diluted in distilled water (Otsuka water for injection (5 ml), Egypt Otsuka Pharmaceutical Co. S.A.E, Egypt) to yield a 2.5% concentration that is used to prevent systemic complications such as renal toxicity. According to their respective sizes, 1.5–3 ml of the solution was injected slowly into the lesion using a 23-gauge needle until it leaked out from the surface. Then, the lesion was compressed for five minutes and observed daily for a week after injection until it became necrotic and fell off spontaneously. (Fig. 2a-c).

Postoperatively, patients from both groups were prescribed a twice-daily nonsteroidal anti-inflammatory drug, Diclofenac Potassium (Catafast^®^ 50 mg sachets, Novartis, Ireland) for three days. In addition, all the patients were postoperatively instructed and motivated to maintain proper oral care using modified Bass brushing technique of their teeth 3 times daily by supplied extra-soft toothbrush (Oral-B^®^ Ultra-Thin Sensitive Toothbrush Extra Soft, Procter and Gamble business service Canada Company, United States). Besides mouth rinsing by prescribed concentrated 0.2% Chlorhexidine mouthwash (Listerine^®^ mouthwash, McNeil Consumer Healthcare division of Johnson and Johnson) for enhancing gingival condition postoperatively. It was mandatory to instruct the patients to follow strict oral hygiene measures as these lesions can easily recur if the gingiva became inflamed again due to any source of irritation [1].

### Outcome assessment

All patients were evaluated clinically for the following:

Intraoperative bleeding was reported for participants in both groups. It was interpreted as mild (subsided 20 min after applying pressure with gauze), moderate (required haemostatic irrigation) or severe (required suturing and, possibly, vitamin K or an infusion of fresh frozen plasma).
Postoperative pain intensity perceived on the 2^nd^ and 7^th^ days using the ten-point Numeric Rating Scale (NRS) interpreted as 0 grade (No pain), 1–3 grade (mild pain), 4–6 grade (moderate pain) and 7–10 grade (severe pain) [42].
The healing quality index was measured according to Landry’s classification [43], which grades the wound on a scale from 1 to 5, where 1 represents very poor healing and 5 represents excellent healing. It records healing based on tissue colour, bleeding, ulceration, granulation tissue and epithelialisation. The healing process was evaluated through follow-up visits on the 1st, 2nd and 4.th postoperative weeks. (Figs. 1c-f; 2c-f; Table 1)
4.Calibration in healing using Landry’s healing quality index criteria was performed for two examiners prior to the study, inter- and intra-examiner reliability were calculated; and kappa ranged from 0.82–0.88 indicating excellent agreement between examiners and across time [44].
Recurrence: patients were recalled for follow-up on the 3rd, 6th and 9th postoperative months to check for any signs of recurrence.

### Statistical analysis

Categorical data were presented as frequencies and percentages and analysed using Fisher’s exact test for intergroup comparisons and Cochran’s q test followed by multiple pairwise comparisons using McNemar’s test with the Bonferroni correction. Quantitative data were presented as mean values and standard deviations. Normality was checked for all quantitative variables using descriptive statistics, plots (histogram, box and whisker and Q-Q plots), and normality tests. Normally distributed continuous data were analysed using the independent-sample *t* test. Skewed continuous data were analysed using the Mann–Whitney *U* test for intergroup comparisons and Friedman’s test followed by the Nemenyi post hoc test for intragroup comparisons. The threshold for statistical significance was set at *P* ≤ 0.05 for all tests. Statistical analysis was performed with R version 4.1.1 for Windows [45].

## Results

The study was conducted on 20 patients that were randomly and equally allocated to two groups. Males and females were equally represented in the laser-treated group; however, in the control group, there were four (40%) males and six (60%) females, with no statistically significant difference between the two groups (*P* = 0.653). The mean age in the test group was 34.20 ± 5.35 years while in the control group it was 36.50 ± 6.25 years. There mean ages of the participants did not differ significantly between the two groups (*P* = 0.388). (Table 2).

**Table 1:** Landry’s healing quality index

Healing grade	Clinical criteria
Very poor 1	Tissue colour: ≥ 50% of gingiva red Response to palpation: bleeding Granulation tissue: present Incision margin: is not epithelialized, with loss of epithelium beyond the lesion site Suppuration: present
Poor 2	Tissue colour: ≥ 50% of gingiva red Response to palpation: bleeding Granulation tissue: present Incision margin: is not epithelialized, with connective tissue exposed
Good 3	Tissue colour: ≥ 25% and < 50% of gingiva red Response to palpation: no bleeding Granulation tissue: none Incision margin: shows no connective tissue exposed
Very good 4	Tissue colour: < 25% of gingiva red Response to palpation: no bleeding Granulation tissue: none Incision margin: no connective tissue exposed
Excellent 5	Tissue colour: all tissues pink Response to palpation: no bleeding Granulation tissue: none Incision margin: no connective tissue exposed

**Table 2:** Summary of statistics of demographic data (sex and age)

Parameter			Laser	Sclerotherapy	*P *value
Sex	Male	*n*	5	4	**0.653 ns**
		%	50.0%	40.0%	
	Female	*n*	5	6	
		%	50.0%	60.0%	
Age	Mean ± SD		34.20 ± 5.35	36.50 ± 6.25	**0.388 ns**

*; significant (*p* ≤ 0.05) *ns*; non-significant (*p* > 0.05)

**Fig. 3:**
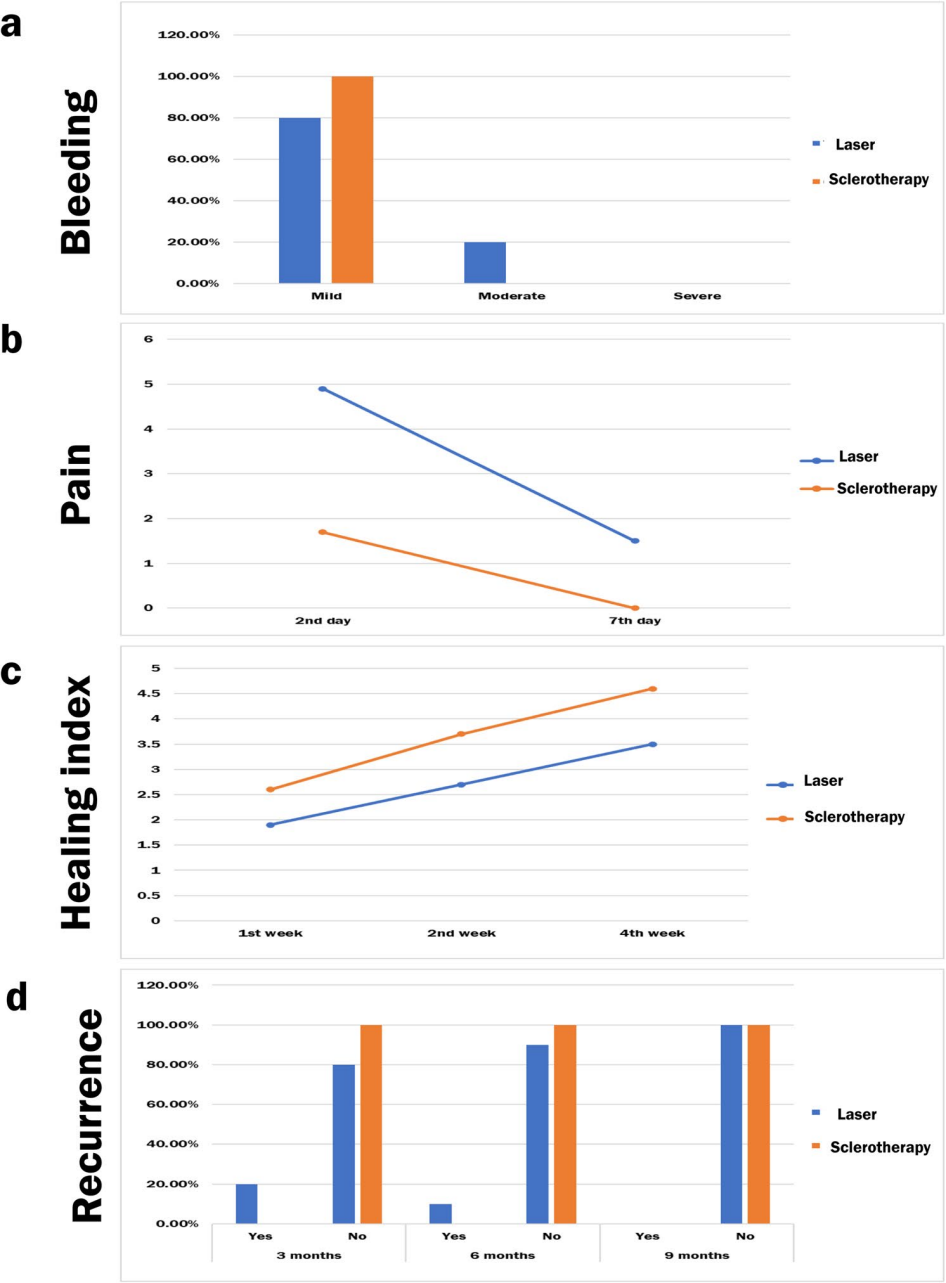
A representative graph showing a comparison between the two groups. **a** Bar chart showing bleeding during surgery. **b** Line chart showing average post-operative pain. **c** Line chart showing the average healing index between the two groups. **d** Bar chart showing recurrence status

The majority (80%) of the participants in the test laser-treated group and all the participants in the control sclerotherapy-treated group had mild bleeding; however, there was no significant difference in the proportions of participants with mild bleeding between the two groups (*P* = 0.481). (Fig. 3a).

On the second and seventh days, the participants in the laser-treated group had significantly more intense postoperative pain than those in the sclerotherapy-treated group (*P* < 0.05). The postoperative pain intensity decreased significantly after the seventh day (*P* = 0.005) for the laser-treated group. For the sclerotherapy-treated group, there was no significant difference between postoperative pain intensity values measured at different time intervals (*P* = 0.066). (Fig. 3b).

Regarding the healing quality index, the sclerotherapy-treated group had higher values at different intervals than the laser-treated group, but the differences were not statistically significant (*P* > 0.05). For both groups, there was a significant difference between values measured at different intervals (*P* < 0.05), with the highest value measured after 4 weeks and the lowest value measured after the first week. Post hoc pairwise comparisons for both groups showed values measured after 4 weeks to be significantly higher than those measured after 1 week (*P* < 0.001). (Fig. 3c; Table 3)

**Table 3:** Mean and Standard deviation (SD) values for healing index for different groups

Time	Healing index (Mean ± SD)		*P value*
	Laser	Sclerotherapy	
1st week	1.90±0.99^**b**^	2.60±1.07^**a**^	**0.137ns**
2nd week	2.70±1.16^**ab**^	3.70±1.06^**ab**^	**0.060ns**
4th week	3.50±1.51^**a**^	4.60±0.52^**a**^	**0.068ns**
*P* value	0.003*	<0.001*	

Different superscript letters indicate a statistically significant difference within the same vertical column *; significant (*p* ≤ 0.05) *ns*; non-significant (*p*>0.05)

Recurrence occurred in two participants in the laser-treated group after three months and in one participant after 6 months while no participant experienced recurrence in the sclerotherapy-treated group in all intervals, with the difference between the two groups being not statistically significant difference (*P* > 0.05). There was no significant difference between the recurrence statuses at different time intervals for the laser-treated group (*P *= 0.368), while the participants in the sclerotherapy-treated group did not experience a recurrence in any time interval. (Fig. 3d).

## Discussion

Conventional treatment modality for oral PG includes complete excision of the lesion with a cold blade together with thorough curettage of the area, due to its origin from the periodontal ligament cells, to prevent recurrence [10, 11]. In nowadays clinical practice, conservative noninvasive treatment options have been vital consideration for patients. The efficacy of the diode laser with different wavelengths, as one of these noninvasive treatment options, has been documented in the treatment of oral PG [41, 46, 47]. They have a number of distinct advantages, including tissue incision, coagulation during surgery and postoperative benefits [48]. In addition, sclerotherapy has been suggested to be an effective treatment modality [23, 32]. It would be a good treatment choice whenever the lesion is either large or develops in a surgically inaccessible area [24].

Our study aimed to evaluate the effectiveness of the diode laser versus sclerotherapy in the treatment of oral PG. The study was conducted on 20 participants that were randomly and equally allocated to both groups. The differences in sex and age between the two groups were not statistically significant, which allowed for an unbiased analysis of the obtained results. Hence, any changes observed in either group will be attributed mainly to the applied approach.

Regarding bleeding findings, most cases in both groups showed mild bleeding during the procedures, with no statistically significant difference in the proportion of those with mild bleeding between the two groups. In line with the findings of Rai et al. [18], Azma et al. [49] and Kocaman et al. [50] who reported no or mild bleeding during laser therapy. This may be explained by the laser’s haemostatic properties, which enable to contract vascular wall collagen [21, 51]. The contraction results in the constriction of the vessels and haemostasis. As for the laser-treated group, only two participants showed moderate bleeding. On the contrary, Al-Mohaya et al. [46] reported a massive haemorrhage from the surgical area when a 940-nm diode laser was used on a diabetic patient. Similarly, Zaghloul et al. [36] attributed bleeding to the large size of the lesions selected. They attributed the reported moderate-to-severe bleeding during the procedure to the patient’s medical condition and the size of the lesion [2, 52].

The participants in the sclerotherapy-treated group showed mild or no bleeding, which is in agreement with the findings of many studies [23, 24, 53, 54]. We suggested that this can be attributed to the damage caused by the sclerosing agent to the vessels’ endothelium, forming a local thrombus that is connected to the vessel wall, which led to the vessel finally becoming fibrotic [23, 53].

However, the diode laser’s ability to perform painless surgery [17, 55, 56] could be attributed to reduced tissue damage and a shift in neural transmission [51], in addition to the significant reduction of bleeding, meaning sutures are not required [57]. In our study, most patients in the laser-treated group reported moderate-to-sever pain. This can be attributed to the fact that lasers induce tissue necrosis, which results in the release of proinflammatory cytokines such as tumour necrosis factor (TNF)-a, which play critical roles in the mediation of inflammation [58].

Parker et al. [59] mentioned that more short-wavelength lasers such as diode lasers 810, 940 and 980 nm disperse the epithelium and can penetrate 2–6 mm into tissue. The risk of deep penetration can cause thermal damage, leading to pain sensation.

On the other hand, the laser-treated group demonstrated a statistically significant difference on the 7th day, indicating progress in the healing process.

In the sclerotherapy-treated group, the majority of patients experienced no pain on the 2nd day of injection, which is in line with the findings of Fernandes et al. [26] who stated that most (90%) patients experienced no pain after the injection. However, one patient experienced postoperative pain after injection that can either be attributed to the superficial injection of the sclerosant solution due to excessive pressure that causes vascular leakage [25] or to the sclerosant permeating the normal dermis before leaking out [28]. Similarly, Da Silva Barros et al. [60] reported that the concentration dose, gradual intravascular application and dilution of the sclerosing agent were factors to be considered when seeking to prevent necrosis, ulceration and oedema and, accordingly, decrease pain. In addition, pain can be referred to from a related dental pathology [59].

One of the clinical observations in our study was that sclerotherapy might achieve faster healing in a shorter time than laser therapy.

The slower rate of healing and increased inflammatory response observed in the laser-treated group can be explained by the fact that when a laser is used to cut soft tissue, a small amount of thermal damage occurs horizontally and vertically around the incision, as indicated by an area of carbonisation, necrosis and irreversibly altered tissue. In comparison to the healing of a scalpel incision, this tissue damage may result in delayed wound healing [61].

Amaral et al. [62] compared diode laser and scalpel surgery for the treatment of fibrous hyperplasia, noting that laser treatment had superior results in terms of discomfort, haemorrhage and surgery duration. It demonstrated a slower rate of recovery compared to scalpel surgery, which could be due to the associated thermal damage to the tissues and the subsequent higher inflammatory response. Additionally, diode lasers produce more apparent changes in the oral tissues. Although a different intervention was used in the control group, their results were consistent with ours.

Moreover, the applied laser parameters, including the laser's wavelength, power setting (watts), continuous/pulsed mode, pulse duration, pulse frequency and exposure time, are all critical laser parameters that influence the extent of thermal injury to tissues [63]. This is in line with the findings of Isola et al. [21] who reported that lasers could produce superior clinical results in terms of aesthetic outcome, with the added benefit of minimal injury to the targeted gingiva. This can be attributed to the use of the 810-nm diode laser in the pulsed mode, which might decrease the thermal damage produced by diode lasers.

In our study, sclerotherapy showed rapid healing with minimal tissue inflammation and no scar formation, which agrees with the findings of Fernandes et al. [26] and Hong et al. [28], who reported no scarring in their patients treated with EO.

However, one participant showed superficial tissue necrosis, which could be attributed to the excessive amount of the sclerosing agent injected into normal tissue. In addition, when the sclerosing agent is applied at a high pressure, it can either damage peripheral nerves or cause skin necrosis, resulting in scars and aesthetic problems [28, 64].

Follow-up after 3, 6 and 9 months revealed that two participants in the laser group experienced recurrence after three months, while one participant did after six months. During the ninth month, there was no case of recurrence in the sclerotherapy group.

The cases of recurrence in the laser group may have been a result of either the remnant lesion or an already-present irritant [19]. Patients not following oral hygiene instructions can also aid in the recurrence of the lesion [41, 47, 65]. None of the patients in the sclerotherapy group reported recurrence, although some were not competent with the given oral hygiene instructions. This could be because sclerosing agents cause damage to the vessels’ endothelium, which leads to fibrosis [54].

Nonetheless, the current study has some limitations. These include using a convenience sampling technique; however, the majority of clinical trials use convenience sampling in which the examiner screen and enroll accessible participants who meet the inclusion criteria [66]. Another limitation is the relatively small sample size which might have impacted the study power, so further studies with larger sample size are still needed and would allow more solid conclusions to be drawn. Furthermore, studies are also needed to assess the treatment outcomes using different types of lasers and sclerosing agent with different concentrations in comparison with conventional surgical excision.

Within the limitations of this study, we can conclude that sclerotherapy could be an excellent choice for the treatment of PG. Both diode laser treatment and sclerotherapy showed to be efficient noninvasive bloodless treatment approaches for oral PG. However, sclerotherapy is less traumatic, inexpensive and simple to perform besides being less prone to recurrence issues, especially when the lesion is either large or surgically inaccessible.

## Data Availability

All data included in this current study are available from the corresponding author upon request.
